# Assessing the Role of Cyberbiosecurity in Agriculture: A Case Study

**DOI:** 10.3389/fbioe.2021.737927

**Published:** 2021-08-19

**Authors:** Tiffany Drape, Noah Magerkorth, Anuradha Sen, Joseph Simpson, Megan Seibel, Randall Steven Murch, Susan E. Duncan

**Affiliations:** ^1^ Department of Agricultural Leadership and Community Education, Virginia Polytechnic Institute and State University, Blacksburg, VA, United States; ^2^ Center for Advanced Innovation in Agriculture, College of Agriculture and Life Sciences, Virginia Polytechnic Institute and State University, Blacksburg, VA, United States; ^3^ Integrated Security Education and Research Center, Virginia Polytechnic Institute and State University, Blacksburg, VA, United States; ^4^ Department of Food Science and Technology, Virginia Polytechnic Institute and State University, Blacksburg, VA, United States; ^5^ Virginia Agricultural Experiment Station, Virginia Polytechnic Institute and State University, Blacksburg, VA, United States

**Keywords:** cyberbiosecurity, cybersecurity, biosecurity, cyber-physical security, agriculture, food

## Abstract

Agriculture has adopted the use of smart technology to help meet growing food demands. This increased automation and associated connectivity increases the risk of farms being targeted by cyber-attacks. Increasing frequency of cybersecurity breaches in many industries illustrates the need for securing our food supply chain. The uniqueness of biological data, the complexity of integration across the food and agricultural system, and the importance of this system to the U.S. bioeconomy and public welfare suggests an urgency as well as unique challenges that are not common across all industries. To identify and address the gaps in awareness and knowledge as well as encourage collaborations, Virginia Tech hosted a virtual workshop consisting of professionals from agriculture, cybersecurity, government, and academia. During the workshop, thought leaders and influencers discussed 1) common food and agricultural system challenges, scenarios, outcomes and risks to various sectors of the system; 2) cyberbiosecurity strategies for the system, gaps in workforce and training, and research and policy needs. The meeting sessions were transcribed and analyzed using qualitative methodology. The most common themes that emerged were challenges, solutions, viewpoints, common vocabulary. From the results of the analysis, it is evident that none of the participating groups had available cybersecurity training and resources. Participants were uncertain about future pathways for training, implementation, and outreach related to cyberbiosecurity. Recommendations include creating training and education, continued interdisciplinary collaboration, and recruiting government involvement to speed up better security practices related to cyberbiosecurity.

## Introduction

Agriculture continues to adopt new smart technologies that allow for increased and remote monitoring of crops and livestock. The interconnectivity of these technologies within a single farm or production facility and in exchange of data with suppliers and vendors creates unsupervised networks of information. With the adoption of these technologies comes increased risks for cybersecurity attacks on farms and agribusinesses (van der Linden et al., 2020). These attacks have the potential to disrupt food supply chains, damaging the bioeconomy and communities. Protecting agriculture includes both good cybersecurity and biosecurity decisions, critical control points, and human behavior and habits that influence the overall security. The combination of these domains has been coined cyberbiosecurity. While cybersecurity encompasses the protection of any electronic data, systems, networks, etc., cyberbiosecurity is one of its most important applications especially focused towards prevention of illegal intrusions and other activities and safeguard the data, information, and other online resources pertaining to life, medical, health, agricultural and food sciences (Murch and DiEuliis, 2019). Experts in the fields of information technology and life sciences tend to lack training in the other, making it difficult to create policies that encapsulate both (Richardson et al., 2019). Since cyberbiosecurity is such a new concept, there are no traditional training and certification courses available, making it difficult to educate people from secondary and post-secondary education to continuing professional development for employees of organizations.

Protecting agriculture and the food supply chain is a high priority, especially with increasing risk of food insecurity brought on by the Covid-19 pandemic (Laborde et al., 2020) as well as the rapid expansion in the global population. Unfortunately, it is uncommon for farms to have response plans for cyber penetrations (van der Linden et al., 2020) or to recognize the risks associated with corrupted data on decision making. Perceived risk of penetration and perceived benefits from better security are two influential factors for adopting better security habits (Geil et al., 2018). Relatively few people in agriculture have training in cybersecurity or biosecurity, which could lead to poor security practices anywhere in the supply chain. Security in a supply chain is only as effective as the weakest link, giving importance to every party involved. To improve cyberbiosecurity practices across the board, training and certifications must be created for current and future workers.

### Cyberbiosecurity in Food and Agriculture

With development of technologies like the worldwide web, agriculture and food production and processing have been incorporated among the cyber-enabled life sciences technologies. Thus, cyberbiosecurity especially in the food and agriculture sector has been recognized by government agencies, producers, and security experts as the solution to cyber-based threats that could have potentially crippling effects on the nation’s food supply chain (Murch et al., 2018). Growth in smart farming is expected to grow on a global scale to reach nearly 26B (USD) by 2028 and the largest market share is centered in North America (Emergen Research, 2020). Smart technologies, while beneficial, have the potential to be exploited by hackers to disrupt the farms using them and the downstream users relying on the supply chain. Potential risks that are attributed with precision agriculture and smart technologies include: false sensor data, data and machinery access control, and data encryption (i.e., ransomware attacks) (Chi et al., 2017). An exploitation in any of these areas could compromise a farm’s entire production. In 2017, the Department of Defense funded the National Strategic Research Institute at the University of Nebraska, along with Colorado State University and Virginia Tech to begin cyberbiosecurity research in biomanufacturing (Global Biodefense, 2017). Their goal was to create a list of preventative procedures for the industry to follow to reduce vulnerabilities to cyberattacks. Unfortunately, some of the solutions and preventative measures are not ‘one size fits all’ solutions because some producers do not have the means to invest the necessary resources into improving their security (Millet et al., 2017). In order to form better practices, experts and professionals from both agriculture and security have looked to other cybersecurity fields to adapt and adopt their procedures to better fit agriculture.

### Current Cyberbiosecurity Initiatives

Cyber-attacks in agriculture are underreported due to a lack of detection capability in current hardware and software (Cyber Security in UK Agriculture NCC Group, 2020). This feeds into the idea of pulling practices from other fields to better adapt to agriculture. Cyber-attacks became more prevalent during the Covid-19, of which the healthcare organizations were the main victim (Pranggono and Arabo, 2020). The universities and organizations working on Covid-19 vaccine development, modelling, and testing were also very much vulnerable to the cyber-criminals (Muthuppalaniappan et al., 2021). The Covid-19 pandemic has increased the value of digitized biodata due to the research to understand the virus and the development of vaccines and other biological response mechanisms built on biodata. This is followed by a call to action for organizations to reallocate resources into understanding and improving cyberbiosecurity, both for preventing another viral or zoonotic-associated pandemic event, such as we are experiencing with Covid-19, from happening (Mueller, 2020), as well as protecting the integrity of the biological data and the systems in which that data is generated, validated, shared, and used for decisions.

### Areas of Opportunity

A common theme among current literature is a call to action for professionals from cybersecurity and biosecurity to collaborate on ways to bring cyberbiosecurity forward into practice (Duncan et al., 2019; Richardson et al., 2019). There is no education and training for individuals interested in cyberbiosecurity to become specialists compared to cybersecurity and biosecurity. The ideal candidate for a job would possibly have a degree in life sciences, including agriculture and food domains, with additional knowledge and training in cybersecurity. (Richardson et al., 2019). Education and training that are kept relevant for the changing field of security need to be created to teach current and future professionals about cyberbiosecurity practices (Richardson et al., 2019).

### A Case for Research in Cyberbiosecurity in Food and Agriculture

As production agriculture continues to adopt smart technologies to monitor and manage their operations, there is an increase in the amount and severity of vulnerability to these farms. A successful cyberattack could disrupt or lead to the destruction of a harvest, which would not only impact the farmers, but it would also ripple through the food system and impact the consumer (Chi et al., 2017). Smart technology, cyber attackers, and cybersecurity procedures are constantly evolving to fit the current environment (Wolfson and Leung, 2020). With agriculture now having a foot in the cybersecurity sector, it too must be able to adapt to the environment to safely operate. Failure to adapt cybersecurity practices to consider the unique structure and complexities of the agriculture and food system could lead to vulnerabilities that could put food supply chains in jeopardy. Future cyberbiosecurity research would allow for farmers and related companies to protect not only their financial interests but also would protect the food supply chain that people and businesses rely on (Duncan et al., 2019). Research specifically in agricultural cyberbiosecurity has not been an area of focus in literature.

### Research Questions

Three research questions guided this work:

1. What is the current experience that professionals in agriculture and food have related to cyberbiosecurity in their field(s)?
2. In what ways are these professionals addressing new situations related to cyberbiosecurity that are new or novel?
3. What feedback or recommendations did the population have for future work related to cyberbiosecurity?

## Methodology

A focused case study on a quasi-experimental research design was employed, using the population that participated in the conference (Privitera and Ahlgrim-Delzell, 2018) using a qualitative approach with multiple sources of data to increase rigor and reliability (Creswell and Plano-Clark, 2007). A qualitative case study allowed for an in-depth understanding of a bound system (cyberbiosecurity in food and agriculture) and focused on the qualitative feedback from three populations: industries related to food and agriculture, faculty at universities who were investigating cyberbiosecurity, and law enforcement agencies (Yin, 2017). This study sought to understand how professionals knowledgeable about cyberbiosecurity worked with it in their respective professions, issues they worked with as a result of cyberbiosecurity breaches, and how to secure data for their entities and clients to avoid a cyberbiosecurity attack in the future. Participants consented to being recorded via Zoom (Zoom Video Communications Inc., San Jose. CA, Zoom.us) and any by-products created were transcribed and analyzed as part of this conference for research purposes. By-products were chat dialogue boxes in zoom, audio and video recorded meeting sessions, and Google Slides that facilitators used during breakout sessions. Data from approximately 80 participants was collected through audio recordings in Zoom, chat box dialog in Zoom, and Google Slides that were populated during breakout sessions. This case study looked to address the current state of cyberbiosecurity in these fields, what the challenges were to investigating and mitigating cyberbiosecurity threats, and what the participants viewed as areas where more resources and efforts should be in place to avoid future threats and mitigate attacks. Concurrently collecting data while the conference was running provided more opportunities for participants to respond and share their experiences, perceptions, and thoughts on cyberbiosecurity (Creswell and Plano Clark, 2007).

### Data Collection

The data used in this paper were collected from a virtual 2-day workshop (Securing Agriculture, Food, and its Economy with Cybersecurity, 2020) held in Fall 2020 as part of a USDA AFRI-funded project (USDA-NIFA Grant No. 2019-67021-29,956, Accession No. 1019771). The workshop was held online via Zoom which included 8 sessions with a variety of agendas (see Table 1) where national and state level speakers addressed a variety of topics of relevance to securing digital, physical, and biological systems and associated data within the domestic food and agriculture system. Those sessions along with breakout rooms were facilitated by trained coordinators to manage the Zoom meeting and Google Slides to help participants organize thoughts, contribute in multiple ways, and enable all to participate to be inclusive to as many participants as possible. The facilitators conducted small group discussion on leadership skills, helped in populating and managing any google based documents, and provided follow up questions. As this was a virtual workshop, participation within each session varied dramatically and only a count record of participants was obtained for each session, which made it difficult to remain informed about what organizations were represented in each session and whose ‘voice’ was heard and contributed to the data for this report. Data were collected from different sources throughout the conference: Zoom audio, Zoom video, and Google based artifacts. The researchers worked with the conference organizers to set up, organize, and transfer any recorded data from Zoom. Any data generated from breakout sessions was also placed into a shared repository that was available to only the research team.

**TABLE 1:** Session agenda for the Securing Agriculture and Food Economy (SAFE) with Cyberbiosecurity virtual workshop (October 6–7, 2020) and data type used for analyses.

Day	Session topic	Data type used
1	Opening and Keynote–Safeguarding our BioEconomy–the Importance of Securing our Agriculture and Food System	Zoom audio
	Crisis for the Food and Agriculture System–Protecting against a Cyberpandemic	Zoom audio, Session notes
	Convergence and Emergence: Bridging the Digital, Physical, and Biological Systems with Cyberbiosecurity	Zoom audio
		Your View: What are the gaps in our current system?	Zoom audio, Breakout rooms
	2	Rallying Cry: Where do you fit? The Why? Who? How? Discussion	Zoom audio, Session notes
		Crisis Scenarios - Are you prepared?	Zoom audio
Workforce Development: Cyberbiosecurity Skills, Resources, and Technical Support Requirements for an Effective Agriculture and Food System		Zoom audio, Session notes
		Next Steps for Priorities: Formal Collaborations and Partnerships, Policies, Science and Technology, Education and Awareness	Zoom audio, Session notes

### Participants

**FIGURE 1:**
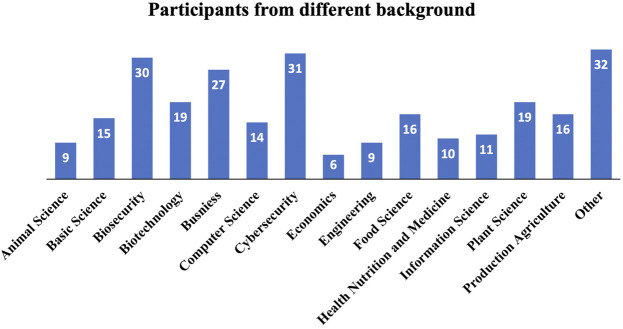
Number of participants within disciplines participating in the Securing Agriculture and Food Economy (SAFE) with Cyberbiosecurity virtual workshop, October 6–7, 2020.

The conference was advertised to a broad audience of professionals involved in food and agriculture, cybersecurity, government security, etc. The university worked with the Virginia Tech office of Continuing and Professional Education to advertise the workshop and register participants for the conference. Advertising included email distributions through a variety of regional and national listservs, LinkedIn posting, Facebook and other social media methods, information on websites, an advance article about the forthcoming workshop published through Virginia Tech news and subsequent national and global media pickup, and word of mouth invitations. Agribusinesses including food manufacturing companies, agricultural producers and commodity boards, auxiliary companies that support the agriculture and food sectors, other private sector businesses representing cybersecurity and technologies, universities, and state and federal agencies including law enforcement, were invited to participate in one or more sessions of the conference. Approximately 170 participants registered for the workshop. Figure 1 represents the number of participants from various backgrounds.

### Data Analysis

*In vivo* coding is a type or category of qualitative data analysis which uses participant’s own words to summarize or analyze the data (Manning, 2017). In this study, *in vivo* was conducted to determine what meaningful patterns were emerging to make up sub-categories of data, based on the conversation and other audio-based recordings and by-products collected (Charmaz, 2006). *In vivo* coding was employed to define patterns in the data and arrange the data in a systematic order (Saldaña, 2021). The data was first open coded where the it was categorized into major themes, and then focused coding was conducted to identify any repeating patterns and understand multi-layer meaning. (Creswell et al., 2007). The resulting codes were more direct and began to explain larger segments of the data as they related to perceptions of cyberbiosecurity. Focused coding helped determine the adequacy of the *in vivo* codes (Charmaz, 2014). By comparing data to data, focused codes were created to help the researcher begin grouping like codes and refining them into larger groups of categories. Focused coding expedited the *in vivo* coding and helped to condense and reorganize what was found in the first round of coding (Charmaz, 2006).

Axial coding was conducted as the final step of the coding process, helping the researchers bring all of the data together and determine themes based on the research questions (Corbin and Strauss, 2008). Development of the codebook emphasized the action-oriented nature of language in which participants discussed the issue of cyberbiosecurity from their viewpoint (Roth, 2008). Using constructs from work presented in the literature review and taking the coding scheme, the codebook was developed around key areas. The quotes in this work have been presented as is without any changes. In order to refrain from making the reading monotonous, all the quotes have not been included in the present article (Anderson, 2010). Only those quotes that best reflect the themes or the findings have been outlined here. The comprehensive data set could be provided upon requesting the corresponding author.

### Limitations

Limitations of this work include the small sample size that is not generalizable to a larger audience about issues related to cyberbiosecurity. The body of literature on cyberbiosecurity related research is still relatively small and reporting baseline data like this can help add to the literature with the knowledge the sample was limited. This work mostly focuses on the current and future cyberbiosecurity situation in the US so had a limited scope on informing cyberbiosecurity issues that might influence international food supply chains. Another limitation is the inability to determine which facets of cyberbiosecurity were left out of the conversations that the data reported. The limits of our current knowledge in this new paradigm is confined to the expertise of the professionals who participated. There is much within the food and agriculture knowledge domains that we do not know or understand that also limits the capacity and potential for cyberbiosecurity characterization and knowledge.

## Results

Through qualitative analysis, four main themes from the conference were identified*: challenges, solutions, viewpoints, and vocabulary*. There is a lack of common language among disciplines that prevents or limits collaboration and communication between disciplines. For some, there has either been a lack of resources or knowledge of resources available for agribusinesses, affiliated companies, agriculture organizations, and farmer/producer to start investing in and improving their cybersecurity practices. It was also noted that lack of government involvement and programs has prevented some from increasing their cyberbiosecurity practices. Similar to other literature, we found a lack of basic cybersecurity training in agriculture to be a limiting factor (Duncan et al., 2019). We expand upon the four primary themes, providing quotes to illustrate and support the sub themes within each.

### Theme 1: Challenges

Challenges related to work in cyberbiosecurity were abundant. At least 213 challenges were coded in the transcripts from the sessions. Since cyberbiosecurity is a newer issue, all participants at the conference, no matter their respective field or expertise, reported a multitude of challenges that had sprouted recently. One of the principal challenges is lack of infrastructure and expertise:

“One of the challenges with small companies is that they simply do not have the infrastructure and they do not have the expertise … we want everything to be cool and quick and available on our iPhone and security is not always at the forefront of earlier adopters.”

The ability to protect against threats was one of the common challenges that was echoed throughout the conference from multiple perspectives. Additionally, other challenges came from the perspectives of different sectors in cyberbiosecurity that included “*the supply chain impact,*” “*ripple effects*,” and feeling “*way behind*” in regard to food and agriculture industries. Participants shared that many employees within organizations felt as though it wasn’t their responsibility to address these issues or tertiary issues around cyberbiosecurity:

“There’s a mindset that this is something that doesn’t involve me. There’s a mindset that I have found in the sector that this is a technology I don’t deal with, so therefore I don’t do that, or I don’t need that.”

Other participants knew and recognized how reliant food and agriculture are on cyber capabilities but also shared that their businesses were way behind in terms of protecting or being proactive against attacks. Some knew the detrimental effects that an attack could have on the supply chain and movement of products to consumers:

“The supply chain impact, you know, stopping something at one point has a ripple effect. Some things back up, and some things go dry.”

Some challenges spanned beyond the scope of what importance cyberbiosecurity holds and questioned the logistics of bringing it to the private sector:

“how do we connect all of the different players in this? How do we connect the stakeholders involved?”

“What federal agency or agencies are going to take the lead in implementing safe cyberbiosecurity practices? Who’s in charge?”

### Theme 2: Solutions

Solutions came in response to challenges discussed among small groups at the conference. Solutions covered multiple parts of the supply chain and reached to different fields involved in cyberbiosecurity.

Cyberbiosecurity is not a one size fits all solution and will need to be adapted for individual circumstances.

“we want to get people away from the idea that anyone thinks they can just buy some off the shelf thing and plug it in and suddenly it will work for them. So what we need is a much larger conversation and so I think that’s all focused around helping people understand what the definition (cyberbiosecurity) is and making sure they have buy into what that definition is.”

Training and classes have been identified as being important; however, it was questioned who would be qualified to lead cyberbiosecurity initiatives:

“If there were not only training but ‘train the trainer’ kind of approaches with, you know validated packages it could be provided to companies obviously gonna make what you can’t be everywhere and train everybody, but you can get standardized you know, training sets.”

In order to get government support and leadership, it was recommended that cyberbiosecurity initiatives need to be brought to the House of Representatives Agriculture Committee:

“if you want to get real leadership on this, we would need to get Collin Peterson (former) chair of the House Ag Committee to add this to one of the hearings and have it be brought up in a hearing you want to create some leadership”.

Since each producer’s cybersecurity situation is unique, there are steps individual businesses can take to help identify their weaknesses, such as red teaming:

“one approach that I thought was always pretty innovative, was like the white hat hacker approach... you actually send the good guys in there to do penetration testing, and you show them before they’ve been hacked, what their vulnerabilities are in a friendly way you say, you know, this is what we found, maybe there’s some tighten up here.”

### Theme 3: Viewpoints

Collaboration among professionals from different fields showed different viewpoints on similar subjects of conversation. Like other industries such as biomedical, biomanufacturing, and chemical production, agriculture contributes to the bioeconomy. The obstacles surrounding agriculture data are similar to obstacles that other fields in the bioeconomy face.

“when we look at this aspect of the cyber data component, what we found is that when you look at the bioeconomy, its vulnerabilities to cyber issues are really not fundamentally different from that you find in other traditional areas, but you might have different outcomes.”

People in agriculture want to be certain that policy makers would have their best interest in mind by helping to protect their company data.

“From an ag perspective, one of the questions is do the people making these policy determinations understand the value and utility of an agricultural company’s data.”

Some were hesitant to rely on government guidance for setting standards for cyberbiosecurity. Cooperation and viewing cyberbiosecurity as a multidisciplinary field would be required.

“on Capitol Hill, legislative bodies, like committees and member offices have historically addressed either cyber, or bio, or ag, or chem, or whatever else we are talking about separately and just because those things are converging does not mean that people on Capitol Hill are converging and they are going to work together to develop and pass legislation to address this risk”.

### Theme 4: Vocabulary

The fourth theme gathered from the conference was a list of vocabulary terms that were considered to be industry specific terms. These terms were used by attendees in one field that others didn’t have a definition for or that may not be common language for other fields. Table 2 lists some of the new terms and their explanation by the participants. The goal of collecting these terms is to be able to define them and create a working lexicon for professionals to use that will help break language barriers for interdisciplinary collaboration.

**TABLE 2:** Vocabulary terms identified as needed defining by some participants and the context in which terms were used during the Securing Agriculture and Food Economy (SAFE) with Cyberbiosecurity virtual workshop (October 6–7, 2020).

Terms	Use during the sessions/Excerpts
Cyber pandemic	*“the crisis for the food and agricultural system focusing on parallel to our current viral pandemic, and we call it the* ***cyber pandemic*** *.”*
Hacktivism	*“And then detailed such threats in some form of insider threats, industrial control systems or malware ransomware, a term I had not noticed called* ***hacktivism*** *, and intellectual property theft,”*
	*“disrupt networks or expose/destroy data to advance a political or social cause”*
Biosecurity	*“So* ***biosecurity*** *is set of measures aimed at preventing the introduction or spread of harmful organisms, in order to minimize risk of transmission of infectious diseases to people, animals and plants caused by viruses, bacteria, or microorganisms.”*
	*“* ***biosecurity*** *is more about when someone tries to purposely do harm in those systems, right.”*
Cybersecurity, information technologies	*“* ***cybersecurity*** *refers to the body of technologies, processes and practices designed to protect networks, devices, programs and data from attack, damaged or overwrite the authorized to access. Cybersecurity may also be referred to as* ***information technologies*** *.”*
Biosafety	*“* ***biosafety*** *is about when something happens accidentally, that causes harm.”*
Threat actors	*“Who are* ***threat actors*** *: disgruntled employees, dissatisfied customers, criminals, foreign terrorist orgs, homegrown violent criminals, domestic extremist groups, industrial opposition”*
Economic espionage	*“steal trade secrets to help anyone other than owner”*
Insider threat	*“exploit credentials or old access to secure areas, sensitive information, etc.”*
	*“current or former employees/contractors, steal IP or destroy data (life work)”*
Ransomware	*“victims in virtually every sector; infection vector (malicious attachment/link), ransom payment typically in bitcoin, FBI does not advocate paying a ransom”*
Agroterrorism	*“deliberate actions by politically motive extremest groups.”*
Hybrid warfare	*“combines traditional and non traditional methods of attacking critical infrastructure”*
Complex attack	*“threats or incidents on social media accusing gov agencies of cover-ups, lack of transparency,”*
Lateral thinking	*“in which you are in one field, but you are applying how you think and what you think, in that field to another field.”*
Continuous monitoring	*“is obviously the idea that, okay, you’re doing things, you’ve got programs in place, but now you have to monitor them, whether they’re, for regulatory purposes, best practices, or to detect active threats and anomalies, changes to your program”*

## Discussion

The previously mentioned sessions included professionals from the university, producers, and security professionals to invoke interdisciplinary collaboration. Bringing professionals together from different fields addresses one of the recommendations commonly found in cyberbiosecurity literature. This allowed for professionals to build networks outside of their field and experience how different fields approach similar situations.

The experience among professionals in the food and agriculture fields is varied but the major theme running through each area of expertise was challenges and solutions. The challenges that were discussed covered current and future challenges in agriculture that may impact food supply chains. Smart farms are not always built with security in mind, meaning their equipment machines and computers may not have up-to-date or any security measures installed to protect their equipment and data. Employees or farms, big and small, do not always have security and threat mitigation training and awareness. This can lead to issues when it comes to protecting data and being alert to potential security penetrations. If penetrations are detected, not everyone has a clear plan of action to take to limit damages and re-secure themselves. There’s a lack of common language between professionals in agriculture and cyberbiosecurity, which was seen in the conferences where participants would have two different meanings for the same terminology. For those who want to improve their practices, there’s either a lack of available resources or known resources to use. Participants questioned whether smaller businesses and farms could afford to invest the money, time, and other resources into upgrading their cyberbiosecurity measures.

Solutions discussed were a combination of recommendations to real and hypothetical problems brought up during the conferences. Developing classes, trainings, and workshops for students and current professionals about cyberbiosecurity was suggested multiple times. Trainings would help people be more cautious of, and better prepared to deal with security related issues. It would also provide ground for developing common language for those working in both industries. Having producers undergo training would allow them to identify weak points in their security and then secure those issues. Companies should create protocols for farmers/producers/agribusinesses to follow if a security penetration is suspected or detected. State and federal government could also establish security standards for smart farm equipment and offer assistance for farms looking to improve their security.

Viewpoints that were identified highlighted attendees’ understandings and perspectives on different concepts that were discussed. Agriculture will increase the amount of data it produces, which helps drive the bioeconomy. The need to prepare and defend agriculture was commonly agreed upon by all attendees. It was mentioned that malicious attacks on agriculture are not something that most people think of or are prepared for. Multiple people mentioned that they felt agriculture is behind in issues related to security and they didn’t trust the nation to protect the food sector. Having the United States Department of Agriculture (USDA) or the Food and Drug Administration (FDA) implement regulations on food processing and processing safety was suggested. However, it was mentioned that government involvement in cyberbiosecurity is sparse because agriculture, biology, and cybersecurity are often addressed as separate entities, not as a system that intertwines and overlaps.

Continued collaboration and work in cybersecurity was another focal point of the conference. One proposed goal was to host a larger, national conference with experts in cybersecurity, agriculture, biology, and in politics. Expanding the reach would bring more voices, expertise, and influencers to the field. Potential partners that would help expand the reach and available resources for cyberbiosecurity, such as the FDA, and FSIS, were mentioned. When individuals were asked how they saw themselves helping cyberbiosecurity in the future, no definite answers were given. Most attendees had an optimistic outlook for the future of cyberbiosecurity but there was no clear vision of the path or what the future of cyberbiosecurity would look like. The lack of a clear vision shows the need for strong leadership to help raise awareness of the importance that every party plays in its success.

Current literature in cyberbiosecurity points out that agriculture is becoming more and more reliant on the capabilities of technology, thus bringing along cybersecurity risks (van der Linden et al., 2020). Also, more resources need to be invested into cyberbiosecurity research and development to prevent large-scale issues from arising (Mueller, 2020). It was widely accepted among participants that agriculture and the cyber-industry are intertwined and will probably be more so in the future. The more reliant on technology agriculture becomes, the more potential vulnerabilities there are in the food supply chain. Increased vulnerabilities make proper security more important to the supply chain. Some participants noted that they didn’t know if their organizations had response plans for cybersecurity or biosecurity issues, or available resources to help prevent them. This supports the calls from literature for the need to invest resources into cyberbiosecurity research and training to better prepare for potential future issues.

## Conclusion

Agriculture is an expanding industry that is increasingly reliant on smart technology for more accurate and efficient farming (Chi et al., 2017; Emergen Research, 2020). In order to protect the U.S. bioeconomy and food supply chain, agriculture needs to adopt cyberbiosecurity practices (Duncan et al., 2019). However, there is a lack of traditional training routes in cyberbiosecurity available for people in agriculture (Richardson et al., 2019). Calls for interdisciplinary collaboration are common among cyberbiosecurity literature (Duncan et al., 2019; Richardson et al., 2019) However, little has been reported in literature about attempting to drive this interdisciplinary collaboration.

As an initial multi-sector foray into the cyberbiosecurity discussion, Virginia Tech hosted a virtual 2-day conference with 170 registered participants across agriculture, cybersecurity, government, and academia to encourage collaboration. The analysis of the workshop discussions identified themes - challenges, solutions, viewpoints, and vocabulary terms–and creates an opportunity for accelerating future discussions and progress on advancing cyberbiosecurity for the agriculture and food system.

To improve cyberbiosecurity practices in agriculture, education and training need to be created for current and future agriculture professionals. Continued interdisciplinary collaboration is needed to close gaps between cybersecurity and biosecurity. Government implemented security standards for agriculture would help speed up the widespread adoption of cyberbiosecurity practices. Cyberbiosecurity will be imperative to the future success and safety of the agriculture supply chain and bioeconomy, as cyber-attacks are not a matter of if but when, and it would beneficial to improve security to prevent successful attacks rather than as a reaction to a successful one.

## Recommendations

Future research and collaboration among professionals across sectors are needed to best improve cyberbiosecurity in agriculture. Education and training were found to be the most pertinent need for all sectors. Workshops and classes need to be developed and offered to current professionals as well as cybersecurity and agriculture students. Gaining buy-in from business and educating producers so they can start learning what they can do to minimize threats, where their data goes, why it’s important to protect it, and how participating in this process can be good for their businesses bottom line, should be an important goal. Training should be tailored to meet the needs of the particular group understanding that the education and training will have to be adaptable and adoptable in order to meet the needs of each audience. The focus of these trainings should be to familiarize attendees with the concept of cyberbiosecurity, how to identify and perceive potential threats, and how to devise methods of handling such threats. Collaboration must continue among sectors in agriculture and cybersecurity. Conferences, such as the one hosted by Virginia Tech, allow structured conversation and networking opportunities among multiple disciplines. Professionals from agriculture, cybersecurity, academia, and government should construct standards for cybersecurity in agriculture. Having the USDA, FSIS, or FDA implement standards for both producers and machine manufacturers would prompt a quicker transition to a cyberbiosecurity-focused industry.

Another recommendation for future work is to examine and track threats and breaches in a more comprehensive manner. While literature and current events are helpful, it’s impossible to understand the breadth and depth of the field since companies are protecting intellectual property and don’t want their breaches to be public knowledge *per se*. Research and training have limits and without actual cases to learn from or anticipate a response to, this will limit the education and training aspect recommended above.

A final recommendation would be to form synergistic collaborations between industry, government, law enforcement and higher education to provide viewpoints from multiple places to become more agile in responding to cyber-attacks and build infrastructure to train future employees for business, government, or law enforcement. Having a common lexicon, gaining a baseline understanding of what the landscape of the field is, and then taking a collaborative approach to approaching it will be paramount for the United States to remain competitive in the field of cyberbiosecurity.

## Data Availability

The original contributions presented in the study are included in the article/Supplementary Material, further inquiries can be directed to the corresponding author.

## References

[B1] Anderson C. (2010). Presenting and Evaluating Qualitative Research. Am. J. Pharm. Educ. 74 (8), 141. 10.5688/aj7408141 21179252 PMC2987281

[B2] Charmaz K. (2014). Constructing Grounded Theory. Thousand Oaks, CA: Sage Publications.

[B3] Charmaz K. (2006). Constructing Grounded Theory: A Practical Guide through Qualitative Analysis. Thousand Oaks, CA: Sage Publications.

[B4] Chi H. Welch S. Vasserman E. Kalaimannan E. (2017). “A Framework of Cybersecurity Approaches in Precision Agriculture. in” proceedings of the ICMLG2017 5th International Conference on Management Leadership and Governance, Dayton, USA, 2–3 March, 2017. Reading, UK: Academic Conferences and Publishing Limited, 90–95.

[B29] Corbin J. Strauss A. (2008). Basics Of Qualitative Research: Grounded Theory Procedures and Techniques Thousand Oaks, CA: Sage Publications, Inc.

[B5] Creswell J. Plano Clark V. (2007). Designing and Conducting Mixed Methods Research. Thousand Oaks, CA: Sage Publications.

[B6] Creswell J. W. Hanson W. E. Clark Plano V. L. Morales A. (2007). Qualitative Research Designs. Couns. Psychol. 35 (2), 236–264. 10.1177/0011000006287390

[B7] Cyber Security in UK Agriculture NCC Group (2020). Available at: https://research.nccgroup.com/wp-content/uploads/2020/07/agriculture-whitepaper-final-online.pdf.

[B8] Duncan S. E. Reinhard R. Williams R. C. Ramsey F. Thomason W. Lee K. (2019). Cyberbiosecurity: A New Perspective on Protecting U.S. Food and Agricultural System. Front. Bioeng. Biotechnol. 7, 63. 10.3389/fbioe.2019.00063 30984752 PMC6450256

[B9] Emergen Research (2020). Smart Farming Market by Farming Type (Livestock Monitoring, Precision Farming, Others), by Offerings (Software, Hardware, Others), and by Application (Livestock Monitoring Application, Precision Farming Application, Others), Forecasts to 2027. Available at: https://www.emergenresearch.com/industry-report/smart-farming-market.

[B10] Geil A. Sagers G. Spaulding A. D. Wolf J. R. (2018). Cyber Security on the Farm: an Assessment of Cyber Security Practices in the United States Agriculture Industry. Int. Food Agribusiness Manage. Rev. 21 (1030-2018-1811), 317–334. 10.22434/ifamr2017.0045

[B11] Global Biodefense (2017). NSRI to Support Defense Research on Emerging Cyberbiosecurity Concerns. Available at: https://globalbiodefense.com/2017/01/05/cyberbiosecurity. (Accessed February 15th, 2021).

[B13] Laborde D. Martin W. Swinnen J. Vos R. (2020). COVID-19 Risks to Global Food Security. Science 369 (6503), 500–502. 10.1126/science.abc4765 32732407

[B14] Millet L. I. Fischhoff B. Weinberger P. J. (Editors) (2017). Foundational Cybersecurity Research: Improving Science, Engineering, and Institutions Washington, DC: The National Academies Press.

[B15] Manning J. (2017). “vivo Coding,” in The International Encyclopedia of Communication Research Methods. Editors Matthes J. (New York, NY: Wiley-Blackwell), 24, 1–2. 10.1002/9781118901731.iecrm0270

[B16] Mueller S. (2021). Facing the 2020 Pandemic: What Does Cyberbiosecurity Want Us to Know to Safeguard the Future? Biosafety and Health 3, 11–21. 10.1016/j.bsheal.2020.09.007 33015604 PMC7518802

[B17] Murch R. DiEuliis D. (2019). Editorial: Mapping the Cyberbiosecurity Enterprise. Front. Bioeng. Biotechnol. 7, 235. 10.3389/fbioe.2019.00235 31632957 PMC6786000

[B18] Murch R. S. So W. K. Buchholz W. G. Raman S. Peccoud J. (2018). Cyberbiosecurity: an Emerging New Discipline to Help Safeguard the Bioeconomy. Front. Bioeng. Biotechnol. 6, 39. 10.3389/fbioe.2018.00039 29675411 PMC5895716

[B19] Muthuppalaniappan M. Stevenson K. BMedSci M. B. Ch. B. Hons) F. H. E. A. (2021). Healthcare Cyber-Attacks and the COVID-19 Pandemic: an Urgent Threat to Global Health. Int. J. Qual. Health Care 33 (Issue 1). mzaa117. 10.1093/intqhc/mzaa117 33351134 PMC7543534

[B20] Pranggono B. Arabo A. (2021). COVID‐19 Pandemic Cybersecurity Issues. Internet Techn. Lett. 4 (2), e247. 10.1002/itl2.247

[B21] Privitera G. J. Ahlgrim-Delzell L. (2018). Research Methods for Education. Thousand Oaks, CA: Sage Publications.

[B22] Richardson L. C. Lewis S. M. Burnette R. N. (2019). Building Capacity for Cyberbiosecurity Training. Front. Bioeng. Biotechnol. 7, 112. 10.3389/fbioe.2019.00112 31297367 PMC6606988

[B23] Roth W.-M. (2008). The Nature of Scientific Conceptions: A Discursive Psychological Perspective. Educ. Res. Rev. 3 (1), 30–50. 10.1016/j.edurev.2007.10.002

[B24] Saldaña J. (2021). The Coding Manual for Qualitative Researchers. Thousand Oaks, CA: Sage Publications.

[B25] Securing Agriculture, Food, and its Economy with Cybersecurity (2020). A Workshop for Preparing Us for Our Future. Available at: https://www.cpe.vt.edu/cyberbiosecurity/program.html. (Accessed July 1, 2021).

[B26] van der Linden D. Michalec O. A. Zamansky A. (2020). Cybersecurity for Smart Farming: Socio-Cultural Context Matters. IEEE Technol. Soc. Mag. 39 (4), 28–35. 10.1109/mts.2020.3031844

[B28] Yin R. K. (2017). Case Study Research and Applications: Design and Methods United States: SAGE Publications.

[B27] Wolfson J. A. Leung C. W. (2020). Food Insecurity during COVID-19: an Acute Crisis with Long-Term Health Implications. Am. J. Public Health 110 (12), 1763–1765. 10.2105/ajph.2020.305953 32970451 PMC7662000

