# Supplementary material for: Assessing the Role of Cyberbiosecurity in Agriculture: A Case Study
Source: Front Bioeng Biotechnol. 2021 Aug 19;9:737927. doi: 10.3389/fbioe.2021.737927 (PMC8416673; doi:10.3389/fbioe.2021.737927)
Supplement: Supplementary file 1 [file DataSheet1.DOCX]

**Supplementary Materials**

| **Examples of Challenges** |
| --- |
| Lack of infrastructure and expertise |
| Ability to protect against threats |
| Supply chain impact |
| Ripple effects |
| Feeling of ‘way behind’ in being proactive against attacks |
| Lack of accountability or responsibility to be involved |
| Logistics of bringing cyberbiosecurity expertise to the private sector |
| **Examples of Solutions** |
| Solutions need to be adapted for individual organizations’ circumstances |
| Training and classes were identified as important |
| Cyberbiosecurity initiatives need to be brought to legislators, including the House of Representatives Agriculture Committee |
| Unique cyberbiosecurity situations suggest that individual businesses can benefit from red teaming to identify their weaknesses |
| **Examples of Viewpoints** |
| Obstacles surrounding agriculture data are similar to obstacles in other fields, such as biotechnology |
| Make certain that policy makers have agriculture’s best interest in mind by helping to protect their company data |
| There is a hesitancy to rely on government guidance for setting standards for cyberbiosecurity. |
| **Examples of Vocabulary** |
| Multiple terms (see Table 2) were identified that needed defining among participants. |
| **Recommendations** |
| Research and collaboration among professionals across sectors are needed to improve cyberbiosecurity in agriculture |
| Education and training are one of the most pertinent needs for all sectors |
| Buy-in across the agriculture and food system is needed to reduce risks of cyberattacks that cripple the food supply |
| Additional cyberbiosecurity conferences will help to cross-training, conversation, and networking |
| A multidisciplinary, multi-functional team of professionals should construct standards for cyberbiosecurity |
| An approach to examine and track threats and breaches in a comprehensive manner is needed |
| Intentional collaborations among industry, government, law enforcement, and higher education are needed to provide multiple viewpoints to facilitate agility in building infrastructure, responding to cyber-attacks, and training. |
